# Effect of Different Surface Treatments on the Micro-Shear Bond Strength and Surface Characteristics of Zirconia: An In Vitro Study

**DOI:** 10.1155/2022/1546802

**Published:** 2022-04-14

**Authors:** Ann Sales, Shobha J. Rodrigues, M. Mahesh, Kishore Ginjupalli, Thilak Shetty, Umesh Y. Pai, Sharon Saldanha, Puneeth Hegde, Sandipan Mukherjee, Vignesh Kamath, Prashant Bajantri, N. Srikant, Ravindra Kotian

**Affiliations:** ^1^Department of Prosthodontics and Crown and Bridge, Manipal College of Dental Sciences, Mangalore, Manipal Academy of Higher Education, Manipal, India; ^2^Department of Dental Materials, Manipal College of Dental Sciences, Mangalore, Manipal Academy of Higher Education, Manipal, India; ^3^Department of Oral Pathology and Microbiology, Manipal College of Dental Sciences, Mangalore, Manipal Academy of Higher Education, Manipal, India

## Abstract

**Purpose:**

To study the effect of different surface treatments on the micro-shear bond strength and surface characteristics of zirconia.

**Methods:**

Two types of zirconia ceramics were tested: opaque (O) and translucent (T). Each type of zirconia was further allotted into four groups based on the type of surface treatment method. The four groups were: control (C), air abrasion with 110 *µ*m Al_2_O_3_ particles (A), etching with Zircos–E Etching solution for 2 hours (E), and a combination of air abrasion and etching (AE). After the surface treatment, all specimens were ultrasonically cleaned and 10 resin cement cylinders were attached to the zirconia discs in each group. A micro-shear bond strength test was performed in a universal testing machine at a crosshead speed of 0.5 mm/min. The fracture surfaces were assessed under a compound microscope. SEM, EDAX, and AFM analyses were done for the zirconia specimens after being subjected to surface treatment. Statistical analysis for the bond strength test was done using the Shapiro–Wilk test, one-way analysis of variance (ANOVA), and Post hoc Tukey test.

**Results:**

The micro-shear bond strength values for the groups were as follows in megapascals (MPa): OC 18.96 (5.54), OA 22.66 (2.51), OE 28.48 (4.50), OAE 28.63 (4.53), TC 22.82 (5.46), TA 25.36 (5.17), TE 28.12 (4.76), and TAE 32.00 (3.47). The one-way analysis of variance (ANOVA) and post hoc Tukey HSD tests were done which showed significant results in the groups. In opaque zirconia, significant differences were seen in the etching and air abrasion with etching groups when compared with the control and air abrasion groups. There was no difference between the etching and air abrasion with etching groups. For translucent zirconia, the only significant difference was seen in the air abrasion with etching group in comparison with the control and air abrasion groups. The mode of failure was majorly adhesive. The surface topography and surface roughness showed significant differences between the groups. The EDAX results showed material loss that occurred due to sandblasting in the air abrasion groups.

**Conclusions:**

Etching with Zircos–E Etching solution significantly increased the bond strength of zirconia to resin cement when compared with other surface treatment methods. In translucent zirconia, the best results can be achieved by combining etching with air abrasion.

## 1. Introduction

Ceramic, which originates from the Greek word *keramos* meaning burnt object, is made by specific heat treatment followed by cooling to form non-metallic, inorganic solids. Ceramics are available in three forms: crystalline, partly crystalline, or non-crystalline. [1] Ceramics composed of zirconium oxide (ZrO_2_) have been known to have many important applications in the medical field. [2–6].

Out of all the zirconia-based materials, yttrium cation-doped tetragonal zirconia polycrystals (Y-TZP) have remarkably higher mechanical properties due to the stabilization of the tetragonal phase. [6] Zirconia with the addition of 3 mol per cent of yttria is known as 3Y-TZP and has two important properties that make it valuable in dentistry. [6] Its mechanical properties are similar to metals but the colour remains similar to that of natural teeth which makes it an esthetic prosthetic material. [6].

3Y-TZP is mainly used in dentistry for the fabrication of dental crowns and fixed partial dentures. The opaque nature of zirconia is one of its major drawbacks. [7] A solution to counteract the opacity of zirconia was the development of translucent zirconia. Translucent zirconia consists of cubic zirconia in a concentration of 50%. This is because zirconia is doped with 5 mol% yttria which leads to the formation of partially stabilized zirconia with both tetragonal and cubic phases. The nature of cubic zirconia is isotropic in various crystallographic directions which makes the zirconia more translucent by reducing the amount of scattered light at the grain boundaries. [8–11].

Close adaptations of restorative margins along with bond durability are important for the success of any ceramic restoration clinically. [12, 13] Recent systematic reviews reveal a high rate of debonding seen in zirconia crowns. [14–19] To enhance the micromechanical bond between the resin cement and zirconia, the intaglio surface of the restoration must undergo surface modification. [20].

While hydrofluoric acid (HF) etching has been successful in roughening the surface of silica-based ceramics and in increasing their wettability, [21–25] HF does not affect altering the surface structure of zirconia due to the absence of glass in the matrix.

Hence, zirconia surface treatment such as Al_2_O_3_ particle air abrasion, tribochemical silica coating, Er: YAG laser, primer application, and silica nanofilm deposition have been used to achieve higher and stable bonding to resin cement. 10-methacryloyloxyidecyl-dihydrogenphosphate (MDP) is a phosphate ester monomer that forms a bond with densely sintered zirconia by chemically reacting with it [26]. The use of airborne particle abrasion with aluminium oxide (Al_2_O_3_) particles followed by the application of MDP in the form of a primer or as a constituent of the cement is generally considered the gold standard for surface modification of zirconia dental restorations. [27] However, airborne particle abrasion has been known to have multiple disadvantages such as the creation of sharp scratches, cracks, grain pull out, and so on. It damages the surface of the ceramic and causes material loss. [28, 29] These defects are sharp and deep which leads to higher stress developing at their crack tips, which turns them into possible crack initiation sites. [30].

Solutions for etching zirconia have been developed, including a solution composed of multiple acids, which can increase zirconia's surface roughness. [31] The zirconia etching solution is known as Zircos–E Etching solution (Bio Den Co., Ltd., Seoul, South Korea) and contains hydrofluoric acid (HF), hydrochloric acid (HCl), sulfuric acid (H_2_SO_4_), nitric acid (HNO_3_), and phosphoric acid (H_3_PO_4_). [32] Zircos–E Etching solution is a patented surface treatment technology that uses ionization to create a microporous surface and improve the bonding strength of zirconia crowns. Because the etching solution treats the total surface area simultaneously, it also increases the bond strength of zirconia crowns to cement. There are limited studies that compare the effect of this etching solution with other surface treatment methods like air abrasion on the bond strength of resin to zirconia. [31, 33] Also, the impact of the etching solution in combination with airborne particle abrasion and the effect of the etching solution on different types of dental zirconia have not been assessed.

The adhesive strength of the bonding systems has been routinely evaluated using shear bond strength tests in the laboratory. [34] Studies evaluating the micro-shear bond strength (*μ*SBS) of zirconia to resin cement after various surface treatments are very scarce.

Therefore, this study evaluates the effect of various surface treatments on the surface microstructure, surface topography, and micro-shear bond strength of opaque and translucent zirconia with resin cement.

The null hypothesis was that there would be no effect of various surface treatments on the surface microstructure, surface topography, and micro-shear bond strength of opaque and translucent zirconia with resin cement.

## 2. Materials and Methods

Zirconia discs of 3Y-TZP and 5Y-TZP with dimensions 18 mm diameter and 4 mm thickness were milled from fully sintered, isostatically pressed zirconia blanks (Jyoti Ceramic Industries Pvt. Ltd., Maharashtra, India). The types of zirconia used in the study are described in Table 1. Depending on the type of surface treatment, the samples were divided into 8 groups as shown in Table 2.

The sample size for this study was calculated based on an earlier study. Cho et al. [31] in their study tested the shear bond strength of zirconia with resin cement after surface treatment with air abrasion, acid etching, and tribochemical silica coating. The results showed superior bond strength in the etching group as compared to the other two groups.

Based on the results of this study, which showed a shear bond strength of 16.15 MPa and 7.09 MPa with a standard deviation of 1.35 in the air abrasion and etching groups, respectively, the required sample in each group is was calculated to be 10 with 5% alpha error, 90% power of the study, and a clinically significant difference of 2 units using the following equation:

(1) $$\begin{array}{c} N=\frac{2{\left(Z_{1-\left(\alpha /2\right)}+Z_{1-\beta }\right)}^{2}\sigma^{2}}{d^{2}}, \end{array}$$

where

- *N* = Sample size
- *Z* = *Z* score
- *a* = Alpha error
- *Z*(1 − (*α*/2)) = *Z* score for the alpha error chosen
- 1-*β* = Power
- *Z*(1 − *β*) = *Z* score for the power chosen
- *σ* = Average standard deviation
- *d* = The minimum difference in the values which will make clinically relevant impact.

### 2.1. Surface Treatment of Zirconia Discs

**For the Control Groups (OC and TC)**: No surface treatment was done.

**For the Air Abrasion Group (OA and TA)**: Each disc was subjected to air abrasion using 110 *µ*m aluminium oxide particles (Hinrivest, Confident Sales, Karnataka, India) for 15 seconds at a distance of 10 mm at 4 bar pressure in the sandblasting machine (Bego Easyblast, Bremen, Germany). [31] After the air abrasion process, the discs were cleaned with an air syringe.

**For the Etching Group (OE and TE)**: The discs were kept immersed in the Zircos–E Etching solution for 2 hours according to the manufacturer's instructions and thereafter rinsed under cold tap water. [31].

**For the Air Abrasion and Etching Group (OAE and TAE)**: The discs are first subjected to air abrasion using 110 *µ*m aluminium oxide particles for 15 seconds at a distance of 10 mm at 4 bar pressure in the sandblasting machine. [31] After the sandblasting, the discs were cleaned with an air syringe and then kept immersed in the Zircos–E Etching solution for 2 hours according to the manufacturer's instructions and thereafter rinsed under cold tap water.

After the surface treatments of the groups, zirconia discs were cleaned in an ultrasonic cleaner (GT Sonic QT Series, GT Ultrasonic Co. Ltd. Shenzhen City, China) with distilled water for 10 minutes.

### 2.2. Surface Microstructure Analysis

#### 2.2.1. Scanning Electron Microscopy (SEM) for Surface Microstructure Analysis

Two discs from each group were chosen randomly for microstructure analysis. For each sample, sputtering was carried out with a gold layer of 10 nm. Following sputtering, the scanning electronic microscope (SEM, Zeiss EVO MA 18; Carl Zeiss, Jena, Germany) was used for surface analysis in all four groups. SEM was supplemented with Energy Dispersive Analysis of X-rays (EDAX, Oxford EDS (X-act), Abingdon, United Kingdom) to analyze the elemental composition of the discs subjected to different surface treatments.

#### 2.2.2. Atomic Force Microscopy (AFM) for Surface Topography Analysis

Two discs from each group were selected randomly for surface roughness measurement using Atomic Force Microscopy (AFM, Innova SPM AFM, Bruker, Massachusetts, USA). Specimens were tested under a non-contact mode utilizing an AFM cantilever with magneto-resistive sensors incorporated in its tip. The measurements were made at three random locations on each disc using a standardized rectangular spot (50 × 50 *μ*m). The average or arithmetic surface roughness (Ra), root mean square value roughness (Rq), and peak height/maximum roughness (highest value − Rmax or *Z*) of the discs were noted as numeric values in nanometers.

### 2.3. Sample Preparation for Micro-shear Bond Strength Test

**Figure 1:**
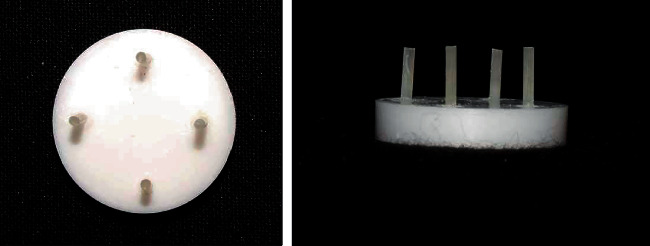
Specimen with resin cement cylinders on zirconia discs.

Silane coupling agent (Silano, Angelus, Londrina, Brazil) was applied to all the surface-treated discs with a micro-brush and gently air-dried followed by 2 coats of adhesive (Single Bond Universal Adhesive, 3M ESPE, St. Paul, Minnesota, USA) which was applied, gently agitated, and dried with a stream for the evaporation of the solvent. Then, according to the manufacturer's instructions, the adhesive was light-cured for 10 seconds. The disc dimensions would only permit 4 samples to be attached per disc. Hence, a total of three discs were used for a total of 10 samples. 4 Tygon tubes with an internal diameter of 0.8 mm and a height of 5 mm were cut for each disc (4 Tygon tubes per disc). Resin cement (RelyX Ultimate, 3M ESPE, St. Paul, Minnesota, USA) was dispensed on a pad and mixed. Each Tygon tube was loaded with cement and placed on the surface of the disc. The four Tygon tubes per disc were positioned at a distance of 5 mm from each other. The samples were light-cured for 40 seconds according to the manufacturer`s instructions. Similarly, three more Tygon tubes were attached to the zirconia disc. A sharp blade was used for the removal of the Tygon tubes from the disc. This resulted in each disc having 4 resin cement cylinders as seen in Figure 1. All the specimens were left to incubate for 24 hours at room temperature for further polymerization.

### 2.4. Micro-shear Bond Strength Test

**Figure 2:**
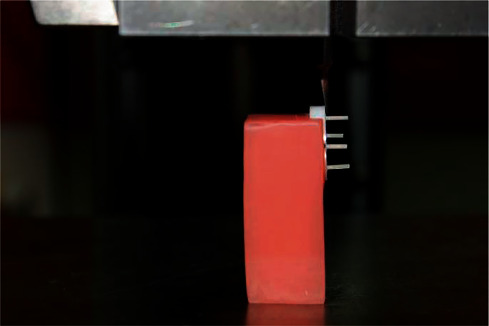
Specimen subjected to Micro-shear Bond Strength (*µ*SBS) Test in the Universal Testing Machine.

The micro-shear bond test involves the application of a loading force using a blade from a universal testing machine to a resin cylinder bonded to a substrate disc. To position the zirconia disc perpendicular to the blade, a heat cure PMMA block of dimension 40 mm × 20 mm × 25 mm was fabricated. It also stabilized the zirconia disc so that the disc would not move during the test. This was then positioned in the universal testing machine (Zwick/Roell Z020, Ulm, Germany). A customized blade (customized and fabricated in Hebich Technical training Institute (HTTI), Mangalore, Karnataka, India) was positioned at an angle of 90° at the junction of the resin/ceramic interface. A shear load was applied to each resin cement cylinder, at a crosshead speed of 0.5 mm/min, until the specimen fractured as seen in Figure 2. The values were recorded as the peak load at failure in Newtons (N). This value was divided by the adhesive surface area (in mm^2^) and the shear bond strength in megapascals (MPa) was obtained.

### 2.5. Mode of Failure Analysis

A compound zoom microscope (Olympus, Olympus Scientific Solutions America Corp, Pennsylvania, USA) was used at 40x magnification to classify the failure mode as adhesive (at the resin cement/zirconia interface), cohesive (within the resin cement or the zirconia), or mixed (with both adhesive and cohesive failures). Two samples for each type of failure in the opaque and translucent zirconia groups were selected and subjected to the scanning electronic microscope (SEM) for analysis of the surface microstructure after testing.

### 2.6. Statistical Analysis

95% confidence interval was used for all statistical tests (*α* = 0.05). Statistical analysis was done with IBM SPSS Statistics software (version 20), Chicago, USA. The distribution of data was assessed using the Shapiro–Wilk test. An independent *t*-test was performed to compare the opaque and translucent zirconia groups in terms of the micro-shear bond strength values. Statistical analysis of the results for the micro-shear bond strength test was performed by one-way analysis of variance (ANOVA) and post hoc Tukey HSD test. A chi-square test was done to analyze the mode of failure for all groups.

## 3. Results

### 3.1. Micro-shear Bond Strength (*µ*SBS) Test

**Figure 3:**
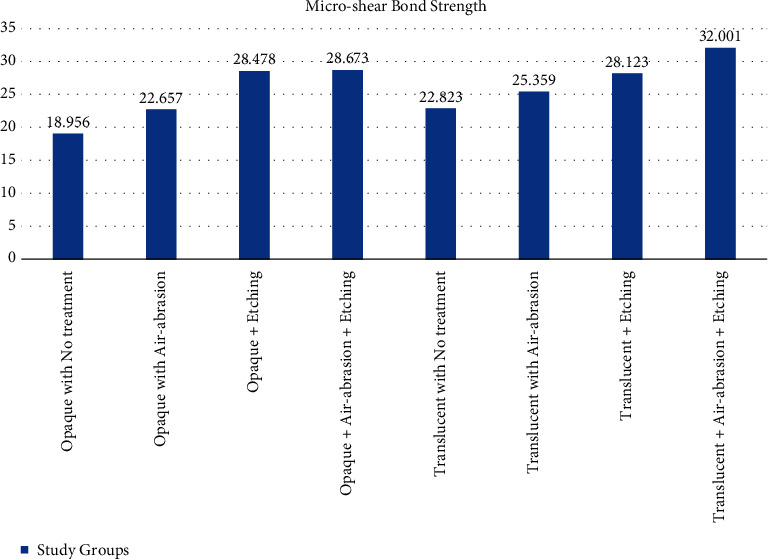
Mean micro-shear bond strength (*µ*SBS) values in MPa for all the groups.

Table 3 shows the mean micro-shear bond strength values (*μ*SBS) and standard deviation with the *F* value of 8.431, *P*-value < 0.001 using one-way ANOVA. The same is represented in Figure 3.

In the opaque group, the *µ*SBS values were highest for the OAE group, followed by the OE group, OA group, and lastly the OC group. In the translucent group, the *µ*SBS values were highest for the TAE group, followed by the TE group, TA group, and lastly the TC group.

### 3.2. Mode of Failure

A compound zoom microscope was used at 40x magnification to classify the failure mode as adhesive or mixed. None of the samples showed cohesive failures.

### 3.3. SEM Analysis

**Figure 4:**
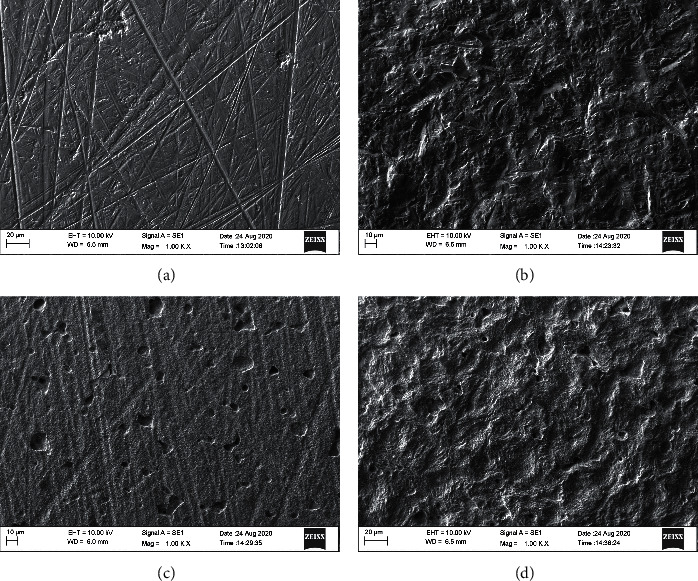
SEM images obtained from each experimental group (Original magnification 1000x). (a) Group OC, (b) Group OA, (c) Group OE, and (d) Group OAE.

**Figure 5:**
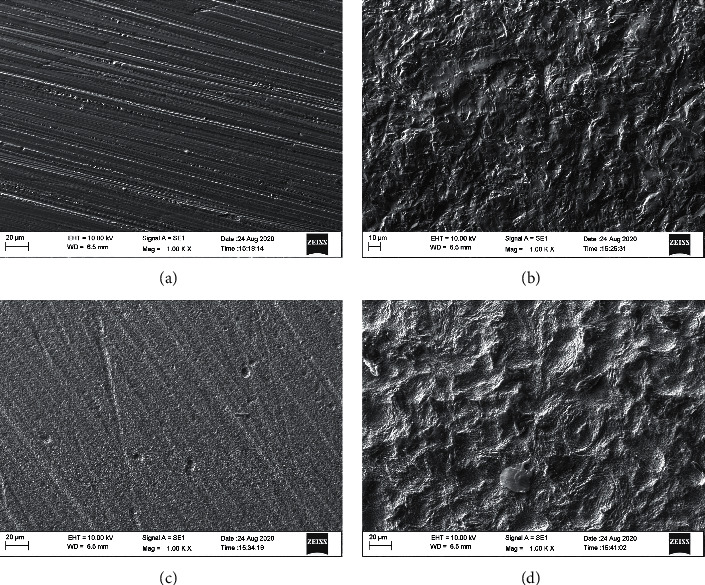
SEM images obtained from each experimental group (Original magnification 1000x). (a) Group TC, (b) Group TA, (c) Group TE, and (d) Group TAE.

Figures 4 and 5 show the results of the SEM analysis at 1000x for the opaque and translucent groups, respectively. For the OC and TC groups, multiple grooves can be seen on the zirconia surface indicating a machined surface. The grooves in the OC groups are much wider and less in number as compared to the TC group. For the OA and TA groups, large irregular peaks and valleys can be appreciated on the zirconia surface. There are no grooves evident. For the OE and TE groups, a uniform coarse appearance can be seen. Faint grooves can be seen on the surfaces. In the OE group, numerous pits can be seen spread out evenly across the coarse surface. For the OAE and TAE groups, a distinct surface can be observed with peaks and valleys along with an erosive appearance. It has characteristics of both air abrasion and etching groups, resulting in a wave-like appearance. Pitting can be appreciated in the OAE group. No grooves are seen in the opaque or translucent zirconia groups.

### 3.4. EDAX Analysis

Table 4 shows the elemental composition in weight percentage (wt%) for all the groups. Aluminium is detected in the OA and TA groups only. Also, the zirconia wt% is relatively lesser in these groups compared to all the other groups.

### 3.5. AFM Analysis

**Figure 6:**
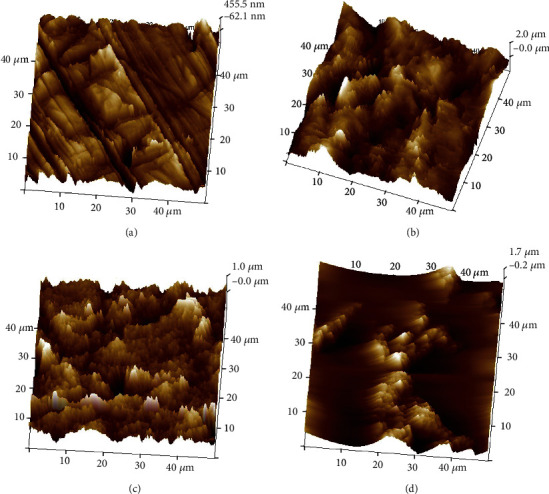
AFM images. (a) Group OC, (b) Group OA, (c) Group OE, and (d) Group OAE.

**Figure 7:**
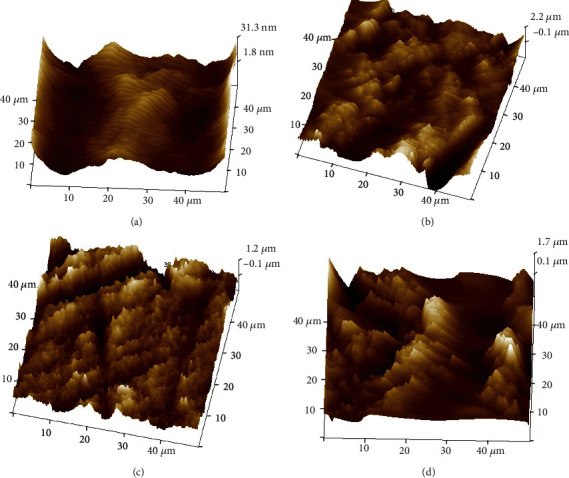
AFM images. (a) Group TC, (b) Group TA, (c) Group TE, and (d) Group TAE.

Figures 6 and 7 depict the 3D images of the AFM analysis of all the groups. The average or arithmetic surface roughness (Ra), root mean square value roughness (Rq), and peak height/maximum roughness (highest value − Rmax or *Z*) are given in Table 5 in nanometers (nm). For both opaque and translucent zirconia, the highest surface roughness is observed in the air abrasion group (OA and TA). The least roughness is seen in the control groups (OC and TC).

### 3.6. Statistical Analysis

The distribution of data was found to be normally distributed using the Shapiro–Wilk test. For all the tests, a significance level of *P*-value ≤ 0.05 was considered. If the calculated *P*-value was less than the threshold, which was chosen for the statistical significance, the null hypothesis was rejected and if the *P*-value was greater than the threshold chosen, then the null hypothesis was accepted.

Comparison of the micro-shear bond strength (*μ*SBS) using the one-way ANOVA test showed that the difference is statistically significant with a *P*-value of <0.001.  Table 6 shows the post hoc Tukey test results for the opaque zirconia groups.  Table 7 shows the post hoc Tukey test for the translucent zirconia groups. Statistical analysis showed that the difference in failure mode was not significant among the groups and that most of the failures were adhesive.

## 4. Discussion

This study was conducted to compare the effect of various surface treatments on the bond strength and surface characteristics of opaque and translucent zirconia. The efficacy of the bond strength of zirconia was evaluated using the micro-shear bond strength test. The effect of different surface treatments on the microstructure of zirconia specimens was evaluated by SEM supplemented with EDAX and AFM topographic analyses. The mode of failure was assessed using a compound microscope.

In recent years, zirconia-based ceramics have been widely used in dentistry, including inlays, crowns, and fixed dental prostheses (FDPs), particularly with the development of dental computer-assisted design/computer-assisted manufacturing (CAD/CAM) systems. [35] Compared to silica-based ceramics, Y-TZP ceramics show difficulty in forming reliable and durable bonds to the resin cement.

To resolve this bonding problem of zirconia restorations, alternative surface treatment methods have been tried using mechanical and chemical approaches. [36, 37] It has been reported that micromechanical bonding using airborne particle abrasion followed by chemical bonding using a 10- methacryloyloxydecyl dihydrogen phosphate (MDP) monomer is effective. [38, 39].

Recently, a zirconia etching solution (Zircos–E Etching solution) has been introduced containing (HF), hydrochloric acid (HCl), sulfuric acid (H2SO4), nitric acid (HNO3), and phosphoric acid (H3PO4). [33] The use of this acidic solution has been reported to increase the bond strength of resin cement to zirconia. [31, 33].

In the present study, the micro-shear bond strength (*µ*SBS) of resin cement with opaque and translucent zirconia was evaluated after subjecting the zirconia specimens to different surface treatment methods: no treatment, air abrasion, etching, and a combination of air abrasion and etching.

In the opaque group, the *µ*SBS values were the highest for the OAE group, followed by the OE group, OA group, and lastly the OC group. Statistical analysis shows that the difference between all the groups is significant except between the OC and OA groups and between the OE and OAE groups. This leads us to assume that air abrasion does not increase the bond strength of opaque zirconia significantly as compared to an untreated surface. Also, etching the opaque zirconia surface will lead to similar bond strength values when compared to air abrasion followed by etching. Hence, etching alone can improve the bond strength of opaque zirconia to resin cement without the need for air abrasion before it.

In the translucent group, the *µ*SBS values were the highest for the TAE group, followed by the TE group, TA group, and lastly the TC group. However, statistical analysis shows only two significant results. The statistically significant results were seen between the TC and TAE groups and between the TA and TAE groups. So, air abrasion with etching of translucent zirconia surface can significantly improve the bond strength as compared to no treatment, or air abrasion or etching surface treatments.

The surface microstructure analysis was done using SEM supplemented with EDAX and AFM. The SEM images at 200x and 1000x revealed significant differences between the groups.

The surface of the OC and TC groups showed a smooth surface interspersed with grooves on the surface. The grooves represent a machined surface. The surface of the OA and TA groups showed irregular roughening of the surface which can be attributed to aluminium oxide sand particles hitting the surface of the zirconia randomly and creating large peaks and valleys with sharp edges on the surface. Grooves were not seen on the surface due to the action of the sand particles.

The OE and TE groups showed a much more uniform treated surface. The surface has no peaks or valleys but had an erosive roughened appearance. The presence of grooves showed that the surface of the zirconia was not aggressively roughened. This is because of the etching solution which chemically treated the zirconia to create micro-porosities on the surface. The surface of the opaque etching group showed multiple pits on the surface along with the erosive surface. The translucent group showed no such surface pitting. The translucent zirconia is more susceptible to etching with acids and the zirconia crystals get leached out uniformly. In the opaque zirconia, since the zirconia crystals are denser, the etching solution leaves pits on the surface of the zirconia due to the non-uniform dissolution of the zirconia.

The OAE and TAE groups showed characteristics of both air abrasion as well as etching groups. Peaks and valleys can be seen on the surface but with an erosive texture as seen in the etching groups. Hence, it has a wave-like appearance.

The EDAX results show that all the specimens consisted of zirconia, oxygen, and hafnium. In the OA and TA groups, the presence of aluminium was also detected which can be due to the remnants of the aluminium oxide sand particles. In OA and TA groups, the weight percentage of zirconia is relatively lesser as compared to the other groups. This further supports the fact that air abrasion aggressively roughens the surface, leading to material loss and damage to the surface of the zirconia.

The AFM analyzes the surface roughness values of the groups. The OC and TC groups had the least values and all the treatment methods increased the surface roughness of the zirconia marginally. The OA and TA groups had the highest roughness values among all the groups. This can be correlated to the SEM analysis; it can be concluded that even though the roughness is higher in the air abrasion group, it is irregular unlike the OE and TE groups and OAE and TAE groups where the roughness is more uniform even if the average roughness value is lesser. Extreme roughness can lead to the weakening of the structure. This is supplemented by the EDAX values which show a lesser zirconia content in the air abrasion groups. Keeping in mind the micro-shear bond strength values, it can be stated that adequate roughness can be achieved with etching which is enough to improve the bond strength. Beyond a certain roughness value, the micromechanical adhesive bond of resin cement to zirconia will not be affected.

The comparison between the opaque and translucent zirconia roughness values shows greater values in the translucent groups. This can be attributed to the fact that translucent zirconia is less dense and more susceptible to surface treatment.

Previous studies assessing the effect of the Zircos–E Etching solution on the bond strength of zirconia showed varying results.

Cho et al. [31] in their study used an older version of the etching solution which was a combination of just hydrofluoric acid and nitric acid. They compared the effect of the etching solution on the interfacial bond strength of zirconia and resin cement as opposed to treatment with air abrasion and tribochemical silica coating. They showed increased bond strength values with the etching group than the other two groups. Their study did not evaluate the combination of air abrasion with etching and they used only one type of zirconia.

Ansari et al. [33] compared the effect of the Zircos–E Etching solution on two different types of zirconia. They divided the samples into four groups: unetched anterior zirconia, etched anterior zirconia, unetched posterior zirconia, and etched posterior zirconia. Anterior zirconia is referred to as translucent zirconia. This study stated that the etching solution increased the bond strength as compared to the unetched groups, but it was significant only in the anterior zirconia group. However, in this study, all the specimens were air abraded before being allotted into the four groups. Hence, the effect of the etching solution alone on the bond strength of zirconia was not evaluated.

Recently, a study done by Zadeh et al. [32] gave contradictory results on the effect of the Zircos–E Etching solution on zirconia. They tested four surface treatment methods of zirconia: air abrasion, etching, air abrasion followed by etching, and etching followed by air abrasion. They reported no significant differences between the groups based on the shear bond strength of the zirconia to the resin cement.

In shear tests, it is very important to understand that the values obtained are mainly useful in comparing and ranking various materials and methods. Hence, the presence of a control group is essential to analyze the results of any laboratory test. None of the previous studies which evaluated the shear bond strength of zirconia after etching with Zircos–E included a control group for reference.

The results of the present study proved that various surface treatments have an effect on the micro-shear bond strength of opaque and translucent zirconia to the resin cement and also affect the microstructure and the surface topography of the opaque and translucent zirconia. Hence, the null hypothesis was rejected.

Etching with the Zircos–E Etching solution significantly increased the bond strength of zirconia ceramics to resin cement when compared with other surface treatment methods. In translucent zirconia, the best results can be achieved by combining etching with air abrasion.

There are certain limitations to the present study. The effect of thermocycling on the bond strength of zirconia after various surface treatments was not assessed. The roughness values were assessed only for two specimens per group. Analysis of all the samples would enable us to establish a better correlation between roughness and bond strength.

Further studies with variations in etching time, the temperature of the etching solution, types of cement, size of the air abrasion sand particles, effect of thermocycling, and the effect of zirconia primers would give us a more detailed insight into the effect of surface treatment of zirconia to improve bond strength.

Other zirconia surface treatments such as Al_2_O_3_ particle air abrasion, tribochemical silica coating, Er: YAG laser, primer application, and silica nanofilm deposition can also be tested and the results can be compared to the conventional treatment methods. Previous studies have evaluated the role of post treatment cleansing on the micro-shear bond strength of lithium disilicate [40]. The effect of various surface cleansing methods can also be evaluated with respect to the micro-shear bond strength of zirconia.

The present report evaluated the bond strength of zirconia. However, as previously conducted for composites, future studies testing other mechanical properties such as hardness [41] and flexural strength [42] should be evaluated in the future also for zirconia-based materials.

## 5. Conclusions

With the limitations of the present study, the following conclusions can be drawn:

- Zircos–E Etching solution can significantly increase the bond strength of opaque zirconia to resin cement as compared to other surface treatment methods.
- Etching of translucent zirconia with the Zircos–E solution after air abrasion results in a significantly higher bond strength value with resin cement as compared to other surface treatment methods.
- SEM and AFM analyses revealed significant differences between different surface treatments. Even though air abrasion provided a rougher surface, it did not necessarily improve the bond strength. Etching creates uniform roughness on the surface and improves bonding.
- EDAX results further proved that air abrasion aggressively roughens the surface and caused material loss.

## Figures and Tables

**Table 1:** Zirconia specimens used in the study.

Type of zirconia	Manufacturer	Composition
Opaque zirconia	Jyoti ceramic industries Pvt. Ltd., Maharashtra, India	Opaque zirconium oxide ceramic disc fully sintered
Translucent zirconia	Jyoti ceramic industries Pvt. Ltd., Maharashtra, India	Isostatically pressed translucent zirconium oxide ceramic disc fully sintered

**Table 2:** Total specimens divided into 8 groups with 10 specimens each.

Type of zirconia	Control	Air abrasion	Etching	Air abrasion + etching
Opaque zirconia	Group 1 (OC)	Group 2 (OA)	Group 3 (OE)	Group 4 (OAE)
Translucent zirconia	Group 5 (TC)	Group 6 (TA)	Group 7 (TE)	Group 8 (TAE)

**Table 3:** Mean micro-shear bond strength (*µ*SBS) values in MPa with standard deviation for all the groups.

Group no.	Group name	Mean *µ*SBS values (sd) (MPa)
1	OC	18.96 (5.54)
2	OA	22.66 (2.51)
3	OE	28.48 (4.50)
4	OAE	28.63 (4.53)
5	TC	22.82 (5.46)
6	TA	25.36 (5.17)
7	TE	28.12 (4.76)
8	TAE	32.00 (3.47)
*F* value -8.431, *P* < 0.001, one way ANOVA		

**Table 4:** Elemental composition in weight % of all the groups.

Group no.	Group name	Zirconia (wt%)	Oxygen (wt%)	Aluminium (wt%)	Hafnium (wt%)
1	OC	69.18	28.81	0	2.01
2	OA	63.48	31.74	2.70	2.09
3	OE	68.58	28.86	0	2.55
4	OAE	67.81	30.07	0	2.12
5	TC	68.86	29.11	0	2.03
6	TA	62.18	32.35	3.23	2.24
7	TE	69.47	28.32	0	2.21
8	TAE	68.50	29.69	0	1.80

**Table 5:** AFM roughness values for all groups at 50 *µ*m in nanometers.

Group no.	Group name	Average roughness Ra (in nm)	Root mean square roughness Rq (in nm)	Maximum roughness rmax (in nm)
1	OC	102	135	1197
2	OA	359	479	4798
3	OE	283	425	3647
4	OAE	317	414	3211
5	TC	8.77	10.8	80.3
6	TA	470	599	4673
7	TE	294	353	2814
8	TAE	336	433	3712

**Table 6:** Post hoc Tukey test for the opaque groups showing significant difference.

Dependent variable	Comparison group	Compared with	Mean difference	Standard error	*P* Value
Micro-shear bond strength (*µ*SBS)	OC	OA	−3.701	1.97209	0.256
		OE	−9.52200	1.97209	**<0.001**
		OAE	−9.67100	1.97209	**<0.001**
	OA	OE	−5.82100	1.97209	**0.027**
		OAE	−5.97000	1.97209	**0.023**
	OE	OAE	−0.149	1.97209	1

**Table 7:** Post hoc Tukey test for the translucent groups showing significant difference.

Dependent variable	Comparison group	Compared with	Mean difference	Standard error	*P* Value
Micro-shear bond strength (*µ*SBS)	TC	TA	−2.536	2.13658	0.639
		TE	−5.3	2.13658	0.08
		TAE	−9.17800	2.13658	**0.001**
	TA	TE	−2.764	2.13658	0.573
		TAE	−6.64200	2.13658	**0.018**
	TE	TAE	−3.878	2.13658	0.283

## Data Availability

The data are available on request to the corresponding author.
